# Supplementary material for: Identification of photoperiod-induced specific miRNAs in the adrenal glands of Sunite sheep (Ovis aries)
Source: Front Vet Sci. 2022 Jul 22;9:888207. doi: 10.3389/fvets.2022.888207 (PMC9354845; doi:10.3389/fvets.2022.888207)
Supplement: Supplementary file 1 [file Data_Sheet_1.ZIP › Supplementary file/miRDeep2 file.zip!/miRDeep2 file/Know_Structure/SPLP42A1/oar-mir-299.pdf]

miRBase precursor : oar-mir-299
Total read count : 216
oar-miR-299-5p read count : 212
oar-miR-299-3p read count : 4
remaining reads : 0

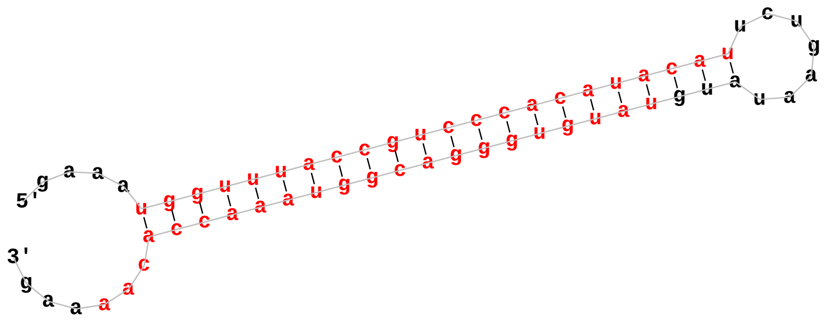

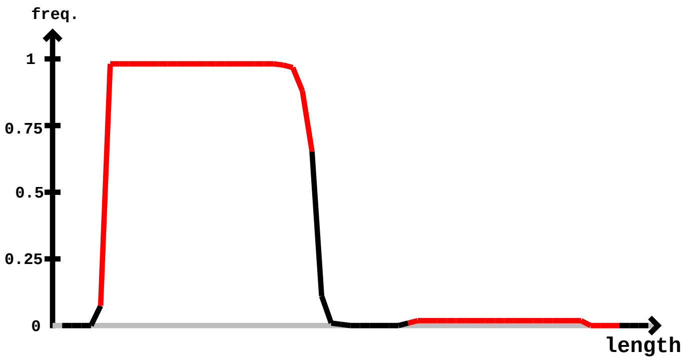


```
       oar-miR-299-5p
                                 oar-miR-299-3p
5'- gaaauggguuuaccgucccacauacauucugaauauguauguggggacgguaaaccacaaaag   -3'    exp
    ....((((((((((((((((((((((.......))))))))))))))))))))))......    reads       mm          sample
    ...augguuuaccgucccacaua..................................    1           0           seq
    ...augguuuaccgucccacauac.................................    4           0           seq
    ...augguuuaccgucccacauaca................................    11          0           seq
    ....ugguuuaccgucccacau...................................    1           0           seq
    ....ugguuuaccgucccacaua..................................    1           0           seq
    ....ugguuuaccgucccacauac.................................    15          0           seq
    ....ugguuuaccgucccacauaca................................    38          0           seq
    ....ugguuuaccgucccacauacau...............................    117         0           seq
    ....ugguuuaccgucccacauacauA..............................    8           1           seq
    ....ugguuuaccgucccacauacauu..............................    14          0           seq
    ....ugguuuaccgucccacauacauuc.............................    1           0           seq
    ....ugguuuaccgucccacauacauucu............................    1           0           seq
    ..................................guauguggggacgguaaacc.......    2           0           seq
    ...................................uauguggggacgguaaacc.......    2           0           seq
```
